# High RIG‐I expression in ovarian cancer associates with an immune‐escape signature and poor clinical outcome

**DOI:** 10.1002/ijc.32818

**Published:** 2019-12-19

**Authors:** Dominik Wolf, Heidi Fiegl, Alain G. Zeimet, Verena Wieser, Christian Marth, Susanne Sprung, Sieghart Sopper, Gunther Hartmann, Daniel Reimer, Maximilian Boesch

**Affiliations:** ^1^ Internal Medicine V Medical University of Innsbruck Innsbruck Austria; ^2^ Medical Clinic III, Oncology, Hematology, Immuno‐Oncology and Rheumatology University Clinic Bonn Bonn Germany; ^3^ Department of Gynecology and Obstetrics Medical University of Innsbruck Innsbruck Austria; ^4^ Department of Pathology Medical University of Innsbruck Innsbruck Austria; ^5^ Tyrolean Cancer Research Institute Innsbruck Austria; ^6^ Institute of Clinical Chemistry and Clinical Pharmacology University Clinic Bonn Bonn Germany; ^7^ Lung Center, Cantonal Hospital St. Gallen St. Gallen Switzerland

## Abstract

Ovarian cancer (OC) is the most lethal gynecological malignancy, with platinum‐based chemotherapy remaining the mainstay for adjuvant treatment after surgery. The lack of indication for immunotherapy may at least in part result from the lack of suitable biomarkers allowing stratification of potentially responding patients. In this monocentric study of 141 cases with OC, we used real‐time quantitative PCR to assess the expression of retinoic acid‐inducible gene‐I (RIG‐I) in primary tumor and healthy ovarian control tissues. RIG‐I expression was correlated to various clinicopathological characteristics as well as to a set of molecular and immunological markers. The prognostic significance of RIG‐I expression was queried in univariate and multivariate analyses and validated in an independent cohort. RIG‐I was overexpressed in the cancerous ovary and correlated with a higher tumor grade. The more aggressive Type‐II cancers and cancers with inactivating p53 mutations exhibited higher RIG‐I expression. RIG‐I levels were also elevated in cancers that recurred after remission or were platinum‐refractory. Survival analyses disclosed RIG‐I as an independent marker of poor outcome in OC. Continuative analyses revealed the molecular and immunological correlates of RIG‐I expression in the tumor microenvironment, including interferon production and a distinct immune‐regulatory signature involving checkpoint molecules (PD‐L1/PD‐1), the RNA‐editing enzyme ADAR1 and the regulatory T cell‐specific transcription factor FoxP3. We conclude that high RIG‐I expression associates with poor outcome in OC, which is explainable by local immunosuppression in the tumor bed. RIG‐I expression may inform checkpoint blockade and/or RIG‐I agonistic targeting in a subset of high‐risk OC patients.

## Abstract 2

**What's new?**

Previous evidence suggests that the antiviral helicase RIG‐I bears tumor‐suppressive activity. The clinical significance of RIG‐I in gynecological cancer remains unclear, however. This single‐center, retrospective, explorative biomarker study of 141 cases with epithelial ovarian cancer (OC) reveals the negative prognostic impact of RIG‐I. RIG‐I is overexpressed in OC tissue, particularly in the more aggressive type‐II OCs, and is an independent prognostic marker for overall survival. RIG‐I high‐expressing tumors carry an interferon signature, linking RIG‐I activation to a cancer microenvironment fostering tumor immune‐evasion including upregulation of immune checkpoints. RIG‐I expression may indicate high‐risk OC patients who may benefit from immunotherapeutic intervention.

**Abbreviations**
FIGO: Fédération Internationale de Gynécologie et d'Obstétrique
ISG: Interferon‐stimulated gene
OC: Ovarian cancer
OS: Overall survival
PFS: Progression‐free survival
RIG‐I: Retinoic acid‐inducible gene‐I
Treg: Regulatory T cell

## Introduction

Ovarian cancer (OC) is the most lethal gynecological tumor and mainly two factors account for this detrimental clinical presentation: (*i*) the lack of early detection methods^1^, ^2^ and (*ii*) the inherently aggressive nature of this malignancy driven at least in part by persistence of chemo‐resistant cancer stem cells and subclonal diversification.^3^, ^4^ Although OC presents as a heterogeneous disease, treatment options are rather uniform and generally involve surgical debulking followed by adjuvant therapy with platinum‐based compounds, frequently in combination with taxanes.^1^, ^2^ Considering the various histological subtypes^5^ and the dualistic pathogenesis that underlies OC,^6^ this is a quite unsatisfactory condition and clearly in conflict with the proclaimed goal of delivering precision medicine. Hence, novel prognostic and/or predictive biomarkers are desirable to refine patient stratification and ultimately tailor treatment for improved outcomes.

*Retinoic acid‐inducible gene‐I* (RIG‐I) is an innate immune receptor helicase facilitating pattern recognition of 5′‐triphosphate RNA produced by viral polymerases upon infection.^7^, ^8^ RIG‐I is broadly expressed in various tissues and cell types (including hematopoietic, epithelial and neuronal cells) and becomes greatly induced after viral encounter.^9^ RIG‐I recognizes different types of RNA viruses^10^ and subsequently initiates downstream antiviral signaling that involves the production of interferons.^9^ Accordingly, mice lacking RIG‐I are highly susceptible to infection with RNA viruses^10^ and similarly, mutational inactivation of RIG‐I confers permissiveness to Hepatitis C viral replication.^11^ Of note, RIG‐I is also implicated in sterile inflammation conditions, such as experimental autoimmune encephalomyelitis, where it acts to restrain tissue damage by negatively regulating T_H_1 and T_H_17 cells.^12^ Thus, although primarily functioning to foster immune responses, particular conditions can elicit more specialized effects of RIG‐I, such as the suppression of certain autoreactive T cell subsets.

Ligand‐mediated activation of RIG‐I signaling triggers apoptotic programs in melanoma cells^13^ and confers protective antitumor immunity mediated at least in part by natural killer cells, dendritic cells and interferons.^14^ In OC cells, targeted activation of RIG‐I leads to MHC class I upregulation and the secretion of proinflammatory mediators, such as IL‐6, CCL5 and TNF‐α.^15^ By this mechanism, OC cells are induced to undergo apoptosis and subsequently become phagocytosed by cocultured monocytes. Authors deduced from these data that RIG‐I activation triggers immunogenic cancer cell death and proposed that this strategy, which ultimately mimics viral infection, might be harnessed for the development of immunotherapeutic approaches against OC.^15^ More recently, RIG‐I activation was shown to promote immunity against pancreatic cancer dependent on TGF‐β1 silencing and CD8+ T cells,^16^ and to be effective in tumor types exhibiting a high degree of cellular heterogeneity, such as glioblastoma.^17^ Stimulation of RIG‐I was also shown to release particular vesicles from melanoma cells that provide on their surface the NKp30 ligand BAT3, thus leading to natural killer cell cytotoxicity and melanoma cell lysis.^18^ In clinical hepatocellular carcinoma samples, low RIG‐I expression is associated with poor survival as well as an unfavorable response to adjuvant therapy with IFN‐α.^19^ Consistently, RIG‐I‐deficient mice are more susceptible to carcinogen‐induced, but not spontaneous, hepatocellular carcinoma formation.^19^

Altogether, plenty of evidence suggests that the antiviral helicase RIG‐I bears tumor‐suppressive activity and that agonistic compounds, such as natural ligands, can confer therapeutic benefit based on tumor cell‐intrinsic (induction of apoptosis) and –extrinsic means (immune engagement). To the best of our knowledge, no data are available regarding the clinical significance of RIG‐I in gynecological cancer. We, therefore, sought to investigate the prognostic value of RIG‐I in OC, trying to address the critical need for improved management of this highly aggressive tumor type. A secondary objective was to find molecular and immunological correlates of RIG‐I expression, thus to delineate a RIG‐I‐associated microenvironmental signature for improved stratification and informed considerations on immunotherapeutic options.

## Materials and Methods

### Sampling of tumor and control specimens

Native tissue samples were systematically accumulated during a period of 19 years (between 1989 and 2008) from epithelial OC patients undergoing primary surgical resection at the Department of Gynecology and Obstetrics at the Medical University of Innsbruck (*n* = 141). Tumor‐free allogenic ovarian or tubal tissue served as control (*n* = 28) and was obtained from cases where (salpingo‐)oophorectomy was indicated for reasons other than malignancy or inflammation. Tissue specimens were frozen under liquid nitrogen immediately after surgery and thenceforth stored at −80°C until the extraction of RNA. The yeast two‐hybrid system was used to detect inactivating/loss‐of‐function mutations in *TP53* (encoding p53) as previously described.^20^

### Characteristics of the study cohort

The study population was dominated by a Caucasian genetic background and comprised all major histological OC subtypes (i.e., serous, mucinous, endometrioid and clear cell) in roughly the frequencies one would expect. Aside from the exclusion of patients with borderline tumors, the study cohort was in no way preselected, and we also did not define an age‐related cut‐off. The average time of follow‐up was 7.60 years (range: 0.08–26.08), in which progression‐free survival (PFS) and overall survival (OS) were defined as follows: the time from surgical debulking to progression or all‐cause death (PFS), and the time from surgical debulking to the last follow‐up or all‐cause death (OS). Staging and grading were conducted based on established *Fédération Internationale de Gynécologie et d'Obstétrique* (FIGO) and WHO criteria, respectively. A majority of patients presented with advanced‐stage disease and a good half of patients exhibited high‐grade serous disease. Almost 90% of patients received adjuvant, platinum‐based chemotherapy and Type‐II cancers were slightly more common than Type‐I cancers. The detailed characteristics of the study population are provided in Table 1. Full medical records and follow‐up information was available for all patients.

**Table 1:** Patient characteristics (*n* = 141)

Parameter	Median	Range
Age (years)	62.42	24.00–88.33
Progression‐free survival (months)	26.00	0.00–292.00
Overall survival (months)	64.00	1.00–313.00
	*n*	%
FIGO stage		
I	28	19.86
II	12	8.51
III	85	60.28
IV	16	11.35
Histological subtype		
High‐grade serous (G2/3)	77	54.61
Low‐grade serous (G1)	2	1.42
Mucinous	41	29.08
Endometrioid	20	14.18
Clear cell	1	0.71
Type‐I/Type‐II cancers^1^		
Type‐I	64	45.39
Type‐II	77	54.61
Tumor grade^2^		
G1	7	5.00
G2	79	56.43
G3	54	38.57
Chemotherapy^3^		
Yes	125	88.65
No	16	11.35

1 Type‐I cancers include low‐grade serous, mucinous, endometrioid and clear cell carcinomas; Type‐II cancers include all high‐grade serous carcinomas.

2 Grading was not available for one patient.

3 Patients received adjuvant, platinum‐based chemotherapy.

Abbreviation: FIGO, Fédération Internationale de Gynécologie et d'Obstetrique.

### Validation cohort

To validate findings from the study cohort (training set), we employed gene expression data from a large, independent cohort of OC patients (test set) publically available at http://www.kmplot.com/ovar.^21^ The tool, termed Kaplan–Meier Plotter,^22^ generates Kaplan–Meier survival plots using underlying TCGA data and supports automated selection of the best cut‐off for two‐group dichotomization.

### RNA isolation and processing

Total RNA was isolated from primary tissue samples using the TRI Reagent® strictly according to the manufacturer's instructions (Sigma‐Aldrich, Seelze, Germany). Residual DNA was eliminated through on‐column DNAse digestion. RNA integrity of all samples was evaluated by assessing the 18S and 28S ribosomal RNA bands in 2% ethidium bromide‐stained agarose gels. Additionally, an RNA integrity score (RIS) was measured in over 60% of all samples by QIAxcel capillary electrophoresis (Qiagen, Hilden, Germany). The RIS corresponds to the RNA integrity number (RIN). In agreement with Fleige and Pfaffl,^23^ samples with a RIS of >5 were used for subsequent gene expression analysis (the median RIS of the samples used in our study was 6). RNA was reverse‐transcribed using the reaction conditions specified in Reimer *et al*.^24^

### Primers and probes

Commercial TaqMan® Assays (Thermo Fisher Scientific, Waltham, MA) were used for ADAR1, ADAR2, EZH2, IFN‐α1, IFN‐α2, IFN‐β, IFN‐γ, PD‐1 and RIG‐I. Primers and probes for FoxP3,^25^ IRF1,^26^ IRF2,^26^ PD‐L1^27^ and TBP were designed using Primer Express™ software (Thermo Fisher Scientific) and synthesized and supplied by Metabion International AG, Planegg, Germany. Assay IDs or primer/probe sequences are given in Supporting Information Table S1. For the sake of better readability, the authors avoid official gene name symbols in the manuscript text and use generic terms instead (e.g., RIG‐I instead of *DDX58*).

### Quantitative real‐time PCR

Amplification of cDNA was performed on a QuantStudio 6 Flex Real‐Time PCR System (Thermo Fisher Scientific). The total reaction volume was 20 μl and the mix contained 10 μl TaqMan® Fast Universal PCR Master Mix, 50 ng cDNA template, 900 nM of sense and antisense primer, respectively, and 250 nM of probe. The reaction conditions were as follows: a denaturing step at 95°C for 20 sec, followed by 40 cycles at 95°C for 1 sec and 65°C for 20 sec. Reactions were performed in triplicate to account for technical variation. Quantification of relative gene expression levels was accomplished using the standard curve method and normalization to TBP.

### Detection of CA 125 in serum

A chemiluminescent microparticle immunoassay was performed for the quantitative determination of CA 125 using the i2000 ARCHITECT system (Abbott, Chicago, IL). CA 125 levels were determined at the time of diagnosis.

### Immunohistochemistry of PD‐L1

Immunohistochemical analyses were conducted using the BenchMark ULTRA automated staining device (Ventana, Oro Valley, AZ/Roche, Vienna, Austria). To this end, formalin‐fixed, paraffin‐embedded tissue samples were prepared and treated with Cell Conditioning Solution (Ventana/Roche). Tissue sections were incubated with the anti‐PD‐L1 monoclonal antibody SP263 (Ventana/Roche) for 30 min at 37°C and detected using the ultraView DAB Detection Kit according to the manufacturer's instructions (Ventana/Roche). Samples were counterstained with hematoxylin, treated with Bluing Reagent (Ventana/Roche), and analyzed under the microscope by a trained and blinded pathologist. Images were acquired on a ProGres® Gryphax Kapella microscope (Jenoptik, Jena, Germany). Tissue specimens from 20 patients were analyzed, in which the specimens were selected based on (*i*) RIG‐I expression by quantitative real‐time PCR (high‐ *vs*. low‐expressing samples to allow for RIG‐I‐stratified PD‐L1 analysis) and (*ii*) availability of the specimens for research. The RIG‐I high‐expressing samples showed an average RIG‐I expression of 27.22 (range: 17.02–52.70) and the RIG‐I low‐expressing samples showed an average RIG‐I expression of 2.62 (range: 1.71–3.36), by quantitative real‐time PCR. Tumor and immune/hematopoietic cells were discriminated based on morphological appearance (marker‐free approach). PD‐L1 expression by tumor cells was evaluated and scored as the percentage of tumor cells with specific membrane staining at any intensity. PD‐L1 expression by immune cells was evaluated and scored as the percentage of tumor‐associated immune cells with specific punctate or membranous staining. Samples were rated PD‐L1‐positive if at least 5% of cells within the field of view expressed PD‐L1. No intensity‐related threshold was defined. Human tonsillar tissue was used as a positive control for PD‐L1 staining.

### Ethical considerations

All patients consented to the use of their biological material for research in written form. The Ethical Review Board of the Medical University of Innsbruck approved our study.

### Statistical analysis

As gene expression values did not follow a Gaussian distribution, statistical analyses were conducted using respective nonparametric tests for two‐ (Wilcoxon–Mann–Whitney method) and three‐or‐more‐group comparisons (Kruskal–Wallis method). In the latter case, post hoc analyses were performed to correct for multiple testing. Results are shown as individual data points with the median value highlighted. The Kaplan–Meier estimator was used to analyze univariate survival along with log‐rank statistical testing. A Cox regression model was established for the multivariate analysis of survival. The final model included RIG‐I expression, age at diagnosis, FIGO stage, tumor grade, residual disease after primary debulking surgery and cancer type (Type‐I *vs*. Type‐II). Statistical analyses were performed using SPSS Statistics 20 (IBM, Armonk, NY). Tests were two‐sided and the nominal significance level was 0.05.

## Results

### Clinicopathological correlates of RIG‐I expression

**Figure 1:**
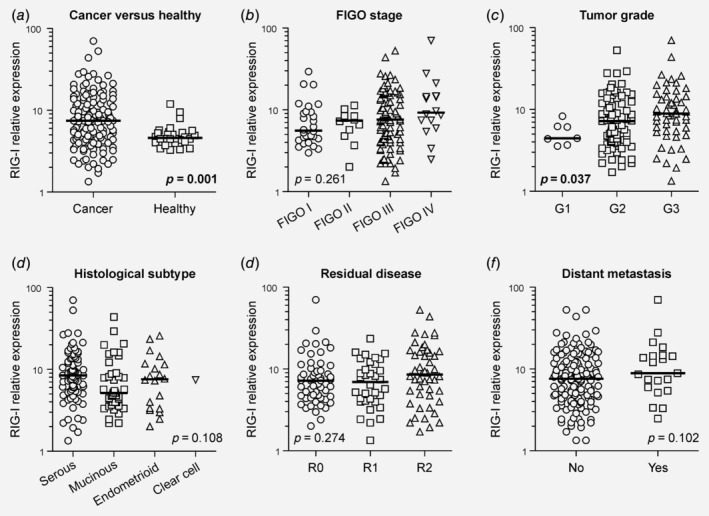
RIG‐I expression associates with clinicopathological characteristics. (*a*) RIG‐I transcript levels were determined using quantitative real‐time PCR and compared among primary OC and healthy ovarian control tissue. (*b–f*) RIG‐I levels were also analyzed subject to various clinicopathological characteristics. (*c*) A post hoc correction for multiple testing yielded nonsignificant results. (*e*) “Residual disease” refers to the resection margin during primary surgical debulking. (*f*) “Distant metastasis” refers to the presence of extra‐peritoneal metastases in organs such as liver, spleen or colon. Abbreviations: FIGO, Fédération Internationale de Gynécologie et d'Obstetrique; OC, ovarian cancer; RIG‐I, retinoic acid‐inducible gene‐I.

To interrogate RIG‐I levels in primary OC and healthy ovarian control tissues, we used quantitative real‐time PCR. We found that RIG‐I levels were significantly higher in the cancerous ovary, suggesting aberrant RIG‐I gene expression during the process of malignant transformation (*p* = 0.001, Fig. 1 *a*). RIG‐I expression tended to be increased in higher FIGO stages (*p* = 0.261, Fig. 1 *b*) and was significantly elevated in higher‐grade tumors (*p* = 0.037, Fig. 1 *c*). Conversely, RIG‐I levels were similar among the principal OC histological subtypes (*p* = 0.108, Fig. 1 *d*) and did not impose a particular outcome of surgical debulking (*p* = 0.274, Fig. 1 *e*). RIG‐I expression also did not associate with the manifestation of extra‐peritoneal metastasis (*p* = 0.102, Fig. 1 *f*). Taken together, RIG‐I is overexpressed in OC and correlates with clinicopathological characteristics.

### RIG‐I associates with Type‐II ovarian cancers

**Figure 2:**
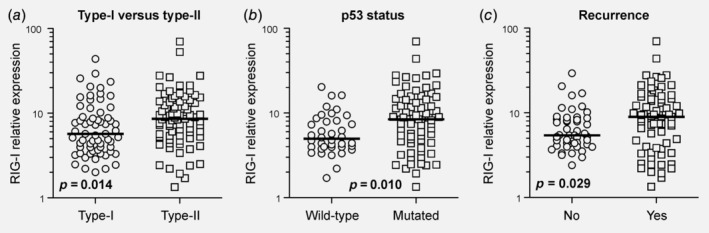
RIG‐I expression correlates with a more aggressive clinical behavior. (*a*) RIG‐I levels were compared among the more indolent Type‐I cancers (low‐grade serous, mucinous, endometrioid and clear cell) and the more aggressive Type‐II cancers (high‐grade serous). (*b*, *c*) RIG‐I levels were also analyzed as a function of inactivating p53 mutations and cancer recurrence. The p53 mutational status was functionally determined using the yeast two‐hybrid system. (*c*) “Recurrence” refers to cancers that recurred after (or progressed under) adjuvant, platinum‐based chemotherapy. Abbreviations: RIG‐I, retinoic acid‐inducible gene‐I.

It has become increasingly clear that ovarian carcinogenesis follows a dualistic model, with Type‐I cancers behaving fundamentally different from Type‐II cancers, both biologically and clinically.^6^ To account for this, we compared RIG‐I levels in Type‐I *vs*. Type‐II cancers, discovering a significant higher RIG‐I expression in the more aggressive Type‐II cancers (*p* = 0.014, Fig. 2 *a*). This finding was further corroborated analyzing RIG‐I expression as a function of the p53 mutational status (*p* = 0.010, Fig. 2 *b*). Confirming the association with a more aggressive clinical phenotype, RIG‐I was expressed at higher levels in cancers that recurred after (or progressed under) adjuvant, platinum‐based chemotherapy (*p* = 0.029, Fig. 2 *c*). Collectively, these data suggest that RIG‐I expression may be linked to OC progression and disease recurrence after remission.

### RIG‐I levels predict ovarian cancer survival

**Figure 3:**
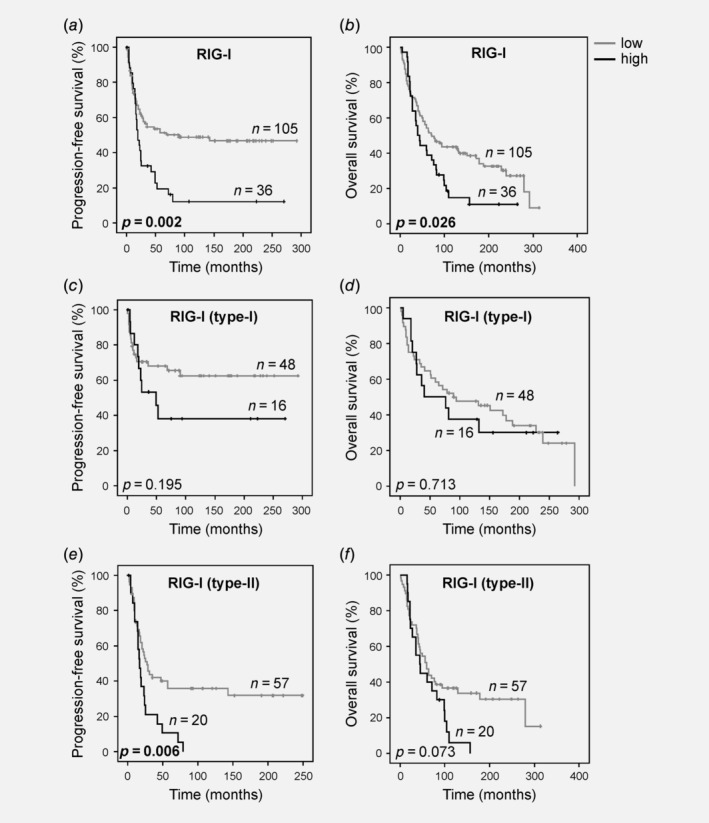
RIG‐I levels predict ovarian cancer outcome. (*a*, *b*) RIG‐I expression was dichotomized according to 75th percentile statistics and PFS and OS were analyzed for the whole cohort using the Kaplan–Meier methodology (*n* = 141). (*c*, *d*) RIG‐I expression was dichotomized according to 75th percentile statistics and PFS and OS were analyzed specifically for Type‐I cancers (low‐grade serous, mucinous, endometrioid and clear cell; *n* = 64). (*e*, *f*) RIG‐I expression was dichotomized according to 75th percentile statistics and PFS and OS were analyzed specifically for Type‐II cancers (high‐grade serous; *n* = 77). Abbreviations: PFS, progression‐free survival; OS, overall survival; RIG‐I, retinoic acid‐inducible gene‐I.

The association of RIG‐I with Type‐II cancers led us to consider prognostic relevance of this innate immune receptor helicase. Strikingly, RIG‐I levels correlated significantly with CA 125, a classical marker of tumor load in OC clinically used for screening or monitoring purpose^1^, ^28^ (*p* = 0.005, Supporting Information Fig. S1). We next performed ROC curve analysis to elaborate a meaningful dichotomization cut‐off for RIG‐I expression to analyze survival (Supporting Information Fig. S2). This investigation revealed that stratification according to 75th percentile statistics (75^lower^/25^upper^) would produce the best separation of two groups. Univariate time‐to‐event analyses using the Kaplan–Meier estimator showed that high RIG‐I expression was associated with both poor PFS (*p* = 0.002, Fig. 3 *a*) and poor OS (*p* = 0.026, Fig. 3 *b*). A separate analysis of Type‐I and Type‐II cancers demonstrated prognostic significance of Kaplan–Meier survival in Type‐II cancers, but not in the more indolent Type‐I cancers which typically lack inactivation mutations in p53^6^ (Type‐I: PFS *p* = 0.195, OS *p* = 0.713, Figs. 3 *c* and 3 *d*; Type‐II: PFS *p* = 0.006, OS *p* = 0.073, Figs. 3 *e and* 3 *f*). To assess whether RIG‐I expression was an independent predictor of OC survival, we set up a Cox regression model which included several parameters known to impact OC outcome. In this multivariate analysis, we disclosed RIG‐I as an independent marker of poor OS, while independent prognostic significance could not be shown for PFS (PFS *p* = 0.107, OS *p* = 0.004, Table 2). Independent prognostic power of RIG‐I expression could also be shown for the subgroup of Type‐II cancers (PFS *p* = 0.051, OS *p* = 0.023, Table 2). Importantly, survival analyses employing mRNA expression data from an independent cohort of OC patients confirmed the prognostic relevance of RIG‐I in this tumor type (Supporting Information Fig. S3). These data establish RIG‐I as a novel biomarker for OC survival.

**Table 2:** Multivariate analysis of survival using a Cox regression model

	Whole cohort (*n* = 141)			
	Progression‐free survival		Overall survival	
Parameter	OR (95% CI)	*p*‐value	OR (95% CI)	*p*‐value
RIG‐I	1.499 (0.916–2.455)	0.107	2.102 (1.265–3.492)	**0.004**
Age^1^	–	–	2.593 (1.653–4.067)	**<0.001**
FIGO stage^2^	1.347 (0.560–3.240)	0.505	1.065 (0.541–2.095)	0.855
Grade^3^	1.549 (0.976–2.456)	0.063	1.373 (0.909–2.075)	0.132
Residual disease^4^	3.240 (1.588–6.610)	**0.001**	2.608 (1.430–4.755)	**0.002**
Cancer type^5^	1.747 (1.063–2.870)	**0.028**	–	–

	Type‐II cancers (*n* = 77)			
	Progression‐free survival		Overall survival	
Parameter	OR (95% CI)	*p*‐value	OR (95% CI)	*p*‐value
RIG‐I	1.819 (0.998–3.315)	0.051	2.025 (1.101–3.722)	**0.023**
Age	–	–	2.365 (1.300–4.305)	**0.005**
FIGO stage	0.847 (0.278–2.581)	0.770	0.927 (0.318–2.709)	0.891
Grade	1.154 (0.668–1.993)	0.607	1.288 (0.742–2.236)	0.369
Residual disease	3.389 (1.382–8.310)	**0.008**	3.209 (1.333–7.726)	**0.009**

Bold *p*‐values refer to statistically significant (*p* < 0.05) results.

1 Age (<median *vs*. >median). Age was not statistically significant in univariate analysis of progression‐free survival (*p* = 0.837), and was therefore not considered in the Cox regression model.

2 FIGO stage (I/II *vs*. III/IV).

3 Grade (G1/G2 *vs*. G3).

4 Residual disease (YES *vs*. NO).

5 Cancer type (Type‐I *vs*. Type‐II). Cancer type was not statistically significant in univariate analysis of overall survival (*p* = 0.277), and was therefore not considered in the Cox regression model.

Abbreviations: CI, confidence interval; FIGO, Fédération Internationale de Gynécologie et d'Obstetrique; OR, odds ratio; RIG‐I, retinoic acid‐inducible gene‐I.

### RIG‐I marks immunosuppression in the tumor microenvironment

**Figure 4:**
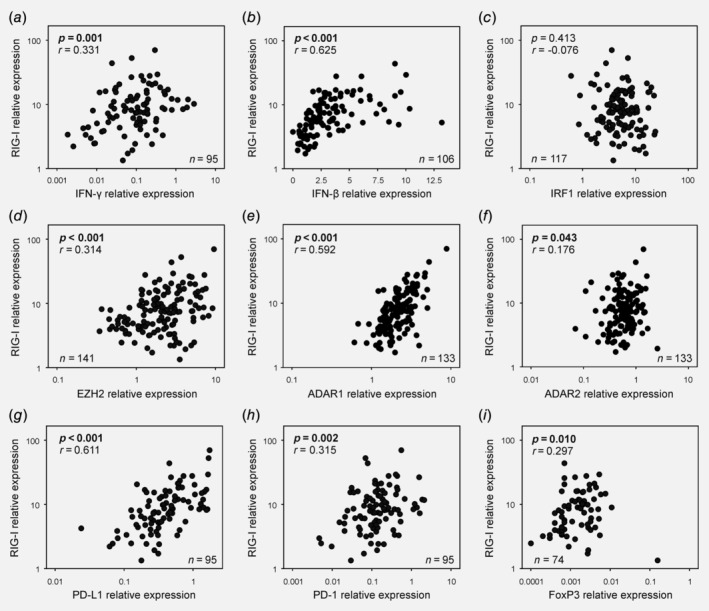
Molecular and immunological correlates of RIG‐I expression. (*a*–*i*) RIG‐I levels were correlated to various molecular and immunological markers which were concomitantly detected in the same ovarian tumor samples using quantitative real‐time PCR. Abbreviations: RIG‐I, retinoic acid‐inducible gene‐I.

**Figure 5:**
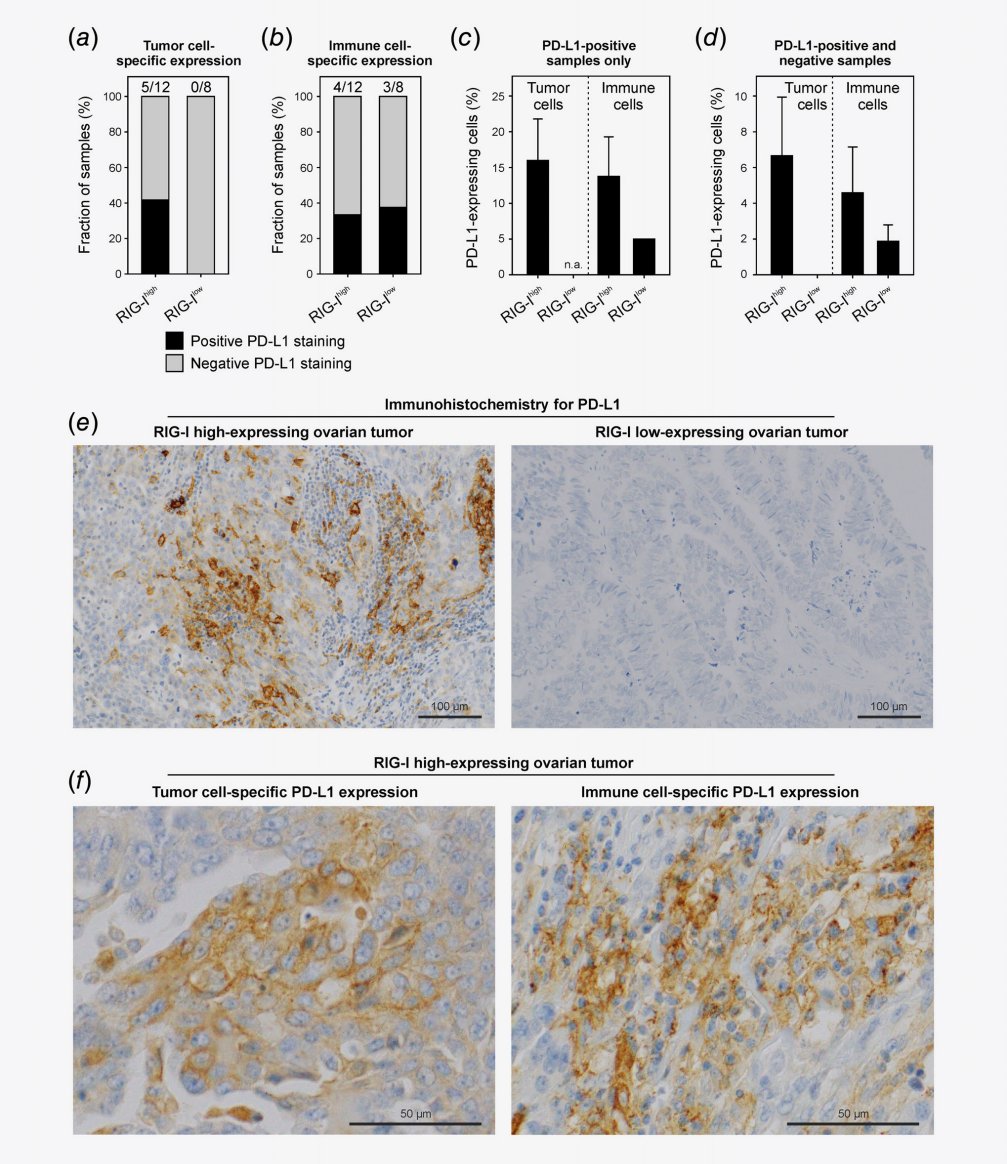
PD‐L1 protein expression in RIG‐I^high^ *versus* RIG‐I^low^ ovarian tumors. (*a*,*b*) Fraction of RIG‐I high‐expressing (*n* = 12) and low‐expressing samples (*n* = 8) staining positive or negative for PD‐L1 in tumor and immune cells, respectively. (*c*) Semi‐quantitative analysis of PD‐L1‐expressing tumor and immune cells in PD‐L1‐positive samples. (*d*) Semi‐quantitative analysis of PD‐L1‐expressing tumor and immune cells in PD‐L1‐positive and PD‐L1‐negative samples (all samples). (*e*) Representative microscopic images of PD‐L1 expression in RIG‐I^high^ *versus* RIG‐I^low^ ovarian tumors. (*f*) Representative microscopic images of PD‐L1 expression in tumor and immune cells. No statistical analysis was performed for this descriptive, corroborative dataset and error bars in (*c*) and (*d*) represent the SEM. Abbreviations: n.a., not applicable; RIG‐I, retinoic acid‐inducible gene‐I; SEM, standard error of the mean.

Considering that literature has quite consistently suggested a tumor‐suppressive function of RIG‐I presumably mediated through interferon‐dependent immune activation, our data appeared paradoxical at first impression. We therefore sought to shed light on the underlying mechanisms by deciphering the molecular and immunological landscape of RIG‐I‐expressing tumors. We found that RIG‐I levels correlated significantly with IFN‐γ (*p* = 0.001, Fig. 4 *a*) and IFN‐β expression (*p* < 0.001, Fig. 4 *b*), indicating pathway activation and feedforward innate immune signaling. However, this was not mirrored by a concomitant increase in interferon regulatory factors, otherwise known to be induced by interferons^29^ (IRF1 *p* = 0.413, IRF2 *p* = 0.722, Fig. 4 *c* and data not shown). Moreover, with the methyltransferase EZH2, RIG‐I levels were tethered to the expression of a proven negative regulator of the RIG‐I pathway^30^ (*p* < 0.001, Fig. 4 *d*). We next investigated the expression of the interferon‐stimulated genes (ISGs) and double‐stranded RNA‐editing enzymes ADAR1 and ADAR2, the former of which is critically required for cancer cell survival in ISG signature‐positive tumors.^31^, ^32^ RIG‐I levels correlated significantly with ADAR1 expression (*p* < 0.001, Fig. 4 *e*) but were less strongly associated with ADAR2 expression (*p* = 0.043, Fig. 4 *f*). This suggested that the innate immune response in ovarian tumors may be antagonized by ADAR1.^33^ To broaden the concept of immune suppression and establish a potential basis for the association of RIG‐I with unfavorable OC outcome, we determined the expression levels of checkpoint molecules^34^ as well as a fate‐specifying transcription factor of regulatory T cells (Tregs).^35^ We found that tumors with high RIG‐I expression were markedly enriched in PD‐L1 (*p* < 0.001, Fig. 4 *g*) and PD‐1 mRNA expression (*p* = 0.002, Fig. 4 *h*). In addition, RIG‐I levels were significantly linked to increased FoxP3 expression, suggestive of tumor infiltration by suppressor cells of the Treg lineage (*p* = 0.010, Fig. 4 *i*). We validated RIG‐I‐associated PD‐L1 expression on protein level using immunohistochemical analysis. We discovered that the fraction of samples with a positive PD‐L1 staining result was higher in the RIG‐I high‐expressing setting, a finding that was also mirrored in a higher percentage of PD‐L1‐positive cells in RIG‐I^high^‐stratified samples (Fig. 5). Interestingly, tumor cell‐specific PD‐L1 expression was only detected in the RIG‐I high‐expressing setting, while PD‐L1 expression in RIG‐I low‐expressing tumors was exclusive to immune/hematopoietic cells.

In sum, these data suggest that the activation of RIG‐I in ovarian tumors is paralleled by a distinct regulatory program involving various mechanisms and pathways, an effect that may limit the innate immune response to a level that no longer constraints, but rather supports, tumor growth.

## Discussion

In this single‐center, retrospective, explorative biomarker study of 141 cases with epithelial OC, we uncovered the prognostic significance of the innate immune receptor helicase RIG‐I, whose canonical function in physiology is to elicit an antiviral response after pattern‐based recognition of viral RNA.^7^, ^8^ Upregulated in the malignant ovary, RIG‐I associated with certain clinicopathological characteristics such as tumor grade, while showing virtual independence from others including histological subtype and postoperative tumor burden. Our results suggested that RIG‐I levels were elevated in more aggressive Type‐II cancers that frequently carry p53 inactivating mutations. Moreover, RIG‐I expression was significantly higher in cancers that relapsed after remission or progressed during adjuvant, cytotoxic treatment with platinum‐based compounds. Accordingly, survival analyses disclosed RIG‐I expression as a marker of disease progression and poor outcome in OC, a finding that could be recapitulated in a large, independent, second validation cohort of OC patients. Thus, our study reports on the identification of a novel OC biomarker with independent prognostic significance.

Of note, our study has three important limitations. First, RIG‐I expression was investigated on the transcript level only, which may not reflect cellular protein abundance,^36^ even though the prognostic power of RIG‐I mRNA expression remains untouched. Second, our study was by scope purely correlative meaning that causal or chronological links cannot be established. Follow‐up investigations employing time‐controlled pharmacological and genetic perturbations are therefore desirable and planned. Third, the healthy controls were largely based on tumor‐free allogenic ovarian (bulk) tissue, with only a minor fraction of samples representing fallopian tube and no samples representing isolated ovarian surface epithelium. Thus, we cannot exclude the possibility that the stromal content of cancer and control tissues were different, even though this would not affect the disclosed prognostic significance of RIG‐I expression.

Given the plethora of published data portending a tumor‐suppressive role of RIG‐I,^13^, ^14^, ^15^, ^16^, ^17^, ^18^, ^19^, ^37^ the results obtained in our study may shed new light on RIG‐I function in cancer and ultimately led to the rejection of our initial working hypothesis (*nota bene*, RIG‐I‐associated tumor control). Specifically, correlation of RIG‐I expression with poor OC survival clearly argues against RIG‐I‐dependent protective immune engagement and/or tumor cell apoptosis in OC. To address this question, we investigated select molecular and immunological features of RIG‐I‐expressing ovarian tumors. Although the association with Type‐I and Type‐II interferons suggested immune activation *in vivo*, the ISGs IRF1 and IRF2^29^ were not coinduced, indicative of suppressed downstream signaling and/or pathway inhibition. In line, EZH2, an epigenetic enzyme known to antagonize the RIG‐I pathway in a methyltransferase‐independent manner,^30^ was positively associated with RIG‐I expression. This observation suggests that in OC, RIG‐I expression (but not necessarily *activity*) is uncoupled from negative EZH2‐control. Further investigations may help to understand the negative prognostic impact of RIG‐I in Type‐II OC, as RIG‐I high‐expressing tumors upregulated various markers suggestive of an immune‐suppressive tumor microenvironment: the immune checkpoints PD‐L1 (CD274) and PD‐1 (CD279)^34^ and the Treg‐specific transcription factor FoxP3^35^ are all linked to high RIG‐I expression. Both immunosuppressive systems (PD‐L1/PD‐1 and Treg infiltration) have previously been shown to predict unfavorable outcome in OC.^25^, ^27^, ^38^ Moreover, RIG‐I levels in ovarian tumors were quite strongly associated with ADAR1 expression, an immune‐silencing factor that may reduce the sensitivity to checkpoint inhibition.^33^, ^39^, ^40^

Taken together, these data suggest that the presumably protective effects of RIG‐I activation and interferon production become neutralized, and finally overridden, by checkpoint‐dependent immunosuppression as well as infiltration with Tregs and direct pathway inhibition through EZH2. The concerted action of these nonredundant mechanisms may abolish tumor immune surveillance, thus fostering tumor growth and imposing a poor outcome.

It is conceivable that antagonized RIG‐I signaling produces a chronic state of low‐level immunity in ovarian tumors that exhausts the anticancer immune response in the presence of persisting antigen.^41^, ^42^, ^43^ Alternatively, upregulation of inhibitory molecules in RIG‐I‐expressing tumors may indicate secondary immune regulation to prevent immunopathology.^44^, ^45^ Following this thought and in view of our data, the interplay of Tregs and the PD‐L1/PD‐1 axis in modulating T cell dysfunction during chronic, viral infection is an interesting side note.^46^ However, it is essentially unclear why RIG‐I becomes upregulated in OC and whether the concomitant detection of an interferon signature is truly reflective of RIG‐I activity and onward signaling, respectively. Indeed, OC development is related to germline BRCA1/BRCA2 mutations, nulliparity, lifetime ovulation cycles and endometriosis,^1^, ^2^, ^6^ while no data have thus far suggested a link with viral infection, even though viral signatures can be found in clinical OC samples.^47^ Thus, if RIG‐I becomes activated in ovarian tumors in the absence of virus, the question arises who is providing the ligand (i.e., 5′‐triphosphate RNA)? One explanation could be that RIG‐I becomes activated through endogenous transposable elements originally stemming from viral species, such as retrotransposons.^48^, ^49^, ^50^ In support of this hypothesis, it has been shown that parts of the effects of cancer therapies depend on RIG‐I activation through small endogenous noncoding RNAs.^51^ Moreover, epigenetic cancer treatment with DNA‐demethylating agents triggers cytoplasmic RNA sensing and viral mimicry that involves endogenous retroviral elements.^52^, ^53^ The expression of multiple endogenous retroviral proteins in OC^54^ may provide a basis for the retrotransposon hypothesis. However, it is also possible that RIG‐I serves RNA‐independent functions in OC (e.g., protein–protein interactions) or that RIG‐I induction is a passenger event with no functional significance for ovarian carcinogenesis whatsoever. Irrespective of the degree of functional involvement, RIG‐I shows upregulation in OC and may serve as a therapeutic target for natural or synthetic ligands. This concept bases on the notion that the provision of high‐dose ligands from an outside source may activate RIG‐I signaling to a greater extent than endogenous ligands in the tumor microenvironment, thus generating high‐level immunity and overcoming immunosuppression and exhaustion.^17^, ^55^ In addition, RIG‐I‐expressing (ISG signature‐positive) tumors may be susceptible to the loss of ADAR1,^31^ which may offer new therapeutic prospects in a subset of patients.

An important aspect of our study concerns the association of RIG‐I expression with the inhibitory checkpoint molecule PD‐L1. While checkpoint blockade has been considered for the treatment of OC,^56^ the clinical success rates have left a lot to be desired so far, which might be a consequence of the relatively low mutational burden of this tumor type. However, given adequate stratification based on predictive biomarkers, checkpoint inhibition may still be a valuable option in OC, particularly as an adjuvant treatment after primary surgical cytoreduction. It remains to be seen whether RIG‐I expression can refine such stratification and inform PD‐L1 targeted therapy in a residual disease setting: while responsiveness to checkpoint inhibition depends at least in part on RIG‐I activation,^57^ RIG‐I associated interferon expression may in turn counteract the efficacy of this immunotherapeutic modality.^58^ In this regard, it is important to note that ISGs expressed by cancer and immune cells are differentially associated with responsiveness to checkpoint inhibition despite their expression being positively correlated (explainable by a threshold phenomenon).^59^ The aggregate of these data suggests that it may be a promising approach to combine checkpoint blockade with RIG‐I agonistic targeting, thus harnessing the interdependence of innate immune pathways and immunotherapies, similarly as shown for the cGAS‐STING pathway in the context of anti‐PD‐L1 treatment in small cell lung cancer.^60^

In summary, we here disclosed RIG‐I as a novel marker for OC progression and poor outcome, particularly in p53‐mutated Type‐II OC. Further investigations suggested that RIG‐I expression was associated with a particular program of immunosuppression involving checkpoint molecules and Tregs, eventually as a consequence of the antecedent interferon response. Overall, our study delivers a new conceptual framework for RIG‐I function in cancer, thus setting the stage for future mechanistic (preclinical) and prospective (clinical) work addressing and tackling RIG‐I‐associated immunosuppression. The combination of checkpoint inhibition and ligand‐mediated RIG‐I activation may represent a promising therapeutic strategy in OC.

## Conflict of interest

G.H. is co‐founder of Rigontec GmbH. The other authors have no potential conflicts of interest to declare. No medical writer or other nonauthor was involved in the preparation of the manuscript.

## Supporting information


**Appendix S1**: Supporting Information

## Data Availability

Data are available from the corresponding authors upon reasonable request.
