# Supplementary material for: High RIG‐I expression in ovarian cancer associates with an immune‐escape signature and poor clinical outcome
Source: Int J Cancer. 2019 Dec 19;146(7):2007–18. doi: 10.1002/ijc.32818 (PMC7028124; doi:10.1002/ijc.32818)

**Supplementary material**

# High RIG-I expression in ovarian cancer associates with an immune-escape signature and poor clinical outcome

Dominik Wolf, Heidi Fiegl, Alain G. Zeimet, Verena Wieser, Christian Marth, Susanne Sprung, Sieghart Sopper, Gunther Hartmann, Daniel Reimer, Maximilian Boesch

## Table of contents

Supplementary tables page 2
    Supplementary table 1 page 2
Supplementary figures page 3
    Supplementary figure 1 page 3
    Supplementary figure 2 page 4
    Supplementary figure 3 page 5

## Supplementary tables

**Supplementary table 1. Primers and probes used for quantitative real-time PCR.**

| *Gene* (alias) | Oligonucleotide | Sequence/assay ID | Manufacturer |
|---|---|---|---|
| ***ADAR* (ADAR1)** | — | Hs00241666_m1 | Thermo Fisher Scientific |
| ***ADARB1* (ADAR2)** | — | Hs00953724_m1 | Thermo Fisher Scientific |
| ***CD274* (PD-L1)** | Sense | 5'-AATGATG GATGTGAAAAAATGTGG-3' | Metabion International AG |
| | Antisense | 5'-AATGCTGGATTACGTCTCCTCC-3' | |
| | Probe | 5'-FAM-TCCAAGATACAAACTCAAAGAAGCAAAGTGATACACATT-TAMRA-3' | |
| ***DDX58* (RIG-I)** | — | Hs00204833 | Thermo Fisher Scientific |
| ***EZH2*** | — | Hs00544833_m1 | Thermo Fisher Scientific |
| ***FOXP3* (FoxP3)** | Sense | 5'-TGGCTAGGAAAATGGCA-3' | Metabion International AG |
| | Antisense | 5'-GCAGGAGCCCTTGTCGG-3' | |
| | Probe | 5'-FAM-TGACCAAGGCTTCATCTGTGGCATCA-TAMRA-3' | |
| ***IFNA1* (IFN-α1)** | — | Hs03044218_g1 | Thermo Fisher Scientific |
| ***IFNA2* (IFN- α2)** | — | Hs00265051_s1 | Thermo Fisher Scientific |
| ***IFNB1* (IFN-β)** | — | Hs00277188_s1 | Thermo Fisher Scientific |
| ***IFNG* (IFN-γ)** | — | Hs00174143_m1 | Thermo Fisher Scientific |
| ***IRF1*** | Sense | 5'-TTTGTATCGGCCTGTGTGAATG-3' | Metabion International AG |
| | Antisense | 5'-AAGCATGGCTGGGACATCA-3' | |
| | Probe | 5'-FAM-CAGCTCCGGAACAAACAGGCATCCTT-TAMRA-3' | |
| ***IRF2*** | Sense | 5'-CGCCCCTCGGCACTCT-3' | Metabion International AG |
| | Antisense | 5'-TCTTCCTATGCAGAAAGCGAAAC-3' | |
| | Probe | 5'-FAM-TTCATCGCTGGGCACACTATCAGT-TAMRA-3' | |
| ***PDCD1* (PD-1, CD279)** | — | Hs01550088_m1 | Thermo Fisher Scientific |
| ***TBP*** | Sense | 5'-CACGAACCACGGCACTGATT-3' | Metabion International AG |
| | Antisense | 5'-TTTTCTTGCTGCCAGTCTGGAC-3' | |
| | Probe | 5'-FAM-TGTGCACAGGAGCCAAGAGTGAAGA-BHQ-3' | |

**Supplementary figures**

**Supplementary figure 1. RIG-I expression correlates with ovarian tumor load.** RIG-I levels were correlated to the tumor marker CA 125, detected on protein level in serum at the time of diagnosis. *Abbreviations used:* RIG-I, retinoic acid-inducible gene-I.

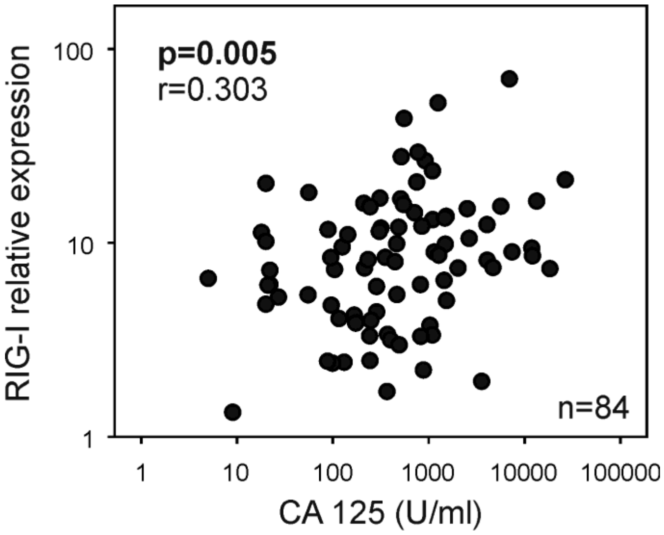

**Supplementary figure 2. Defining an optimal cut-off for survival analysis.** (A+B) ROC curve analysis of RIG-I expression for PFS and OS. (C+D) RIG-I expression was dichotomized according to $50^{th}$ percentile statistics and PFS and OS was analyzed for the whole cohort using the Kaplan-Meier methodology (n=141). (E+F) RIG-I expression was dichotomized according to $25^{th}$ percentile statistics and PFS and OS was analyzed for the whole cohort (n=141). *Abbreviations used:* AUC, area under the curve; PFS, progression-free survival; OS, overall survival; RIG-I, retinoic acid-inducible gene-I; ROC, receiver operating characteristic.

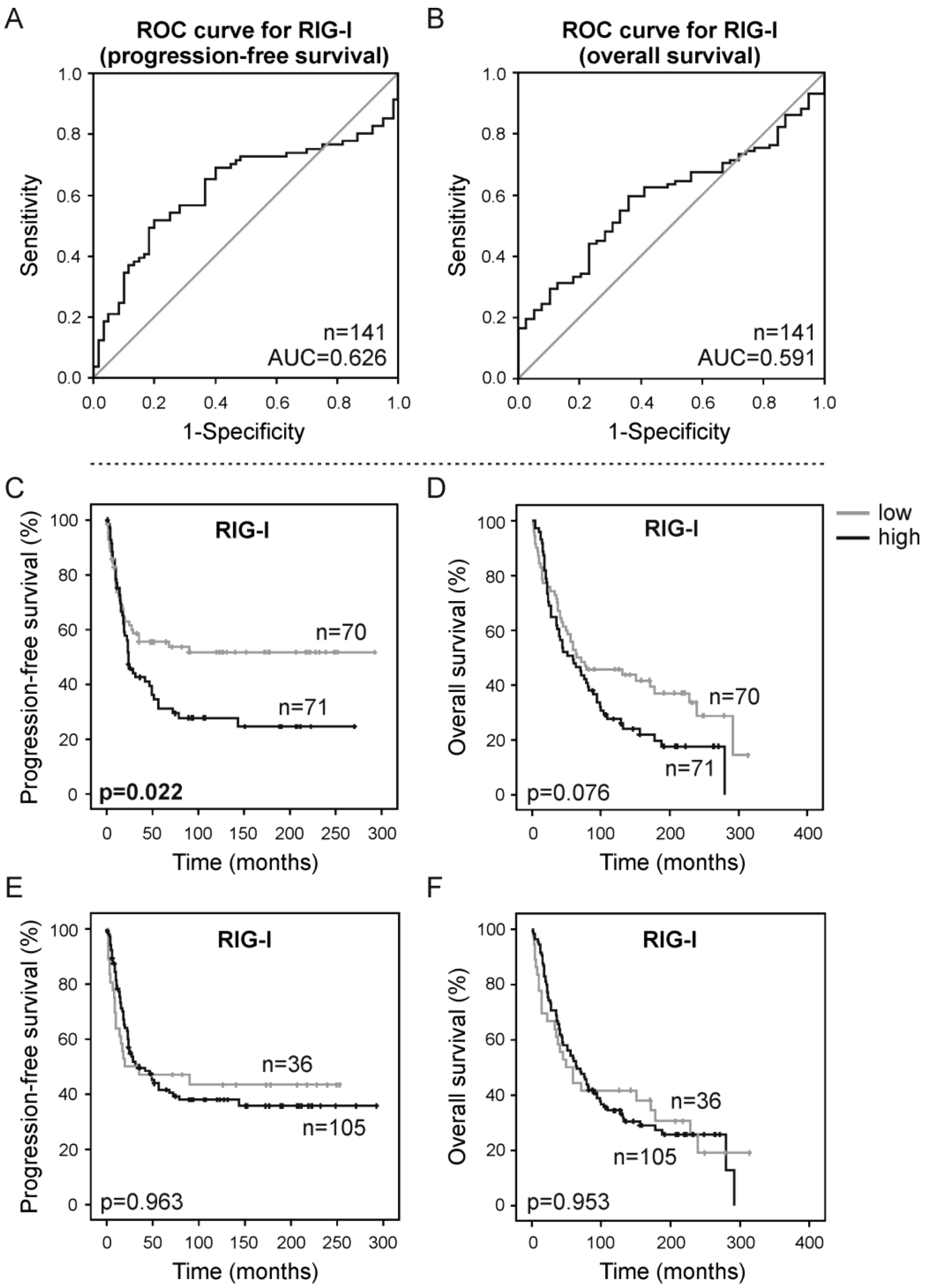

**Supplementary figure 3. Validating the prognostic significance of RIG-I expression.** (A+B) Kaplan-Meier analysis of PFS (n=614) and OS (n=655) in an independent cohort of OC patients using TCGA data available at www.kmplot.com/ovar. RIG-I mRNA expression was detected using a probe with the annotation 222793_at. The algorithm selected the best cut-off for dichotomization (function 'Auto select best cutoff'), which happened to be the 73rd percentile for both PFS and OS. *Abbreviations used:* OC, ovarian cancer; OS, overall survival; PFS, progression-free survival; RIG-I, retinoic acid-inducible gene-I.

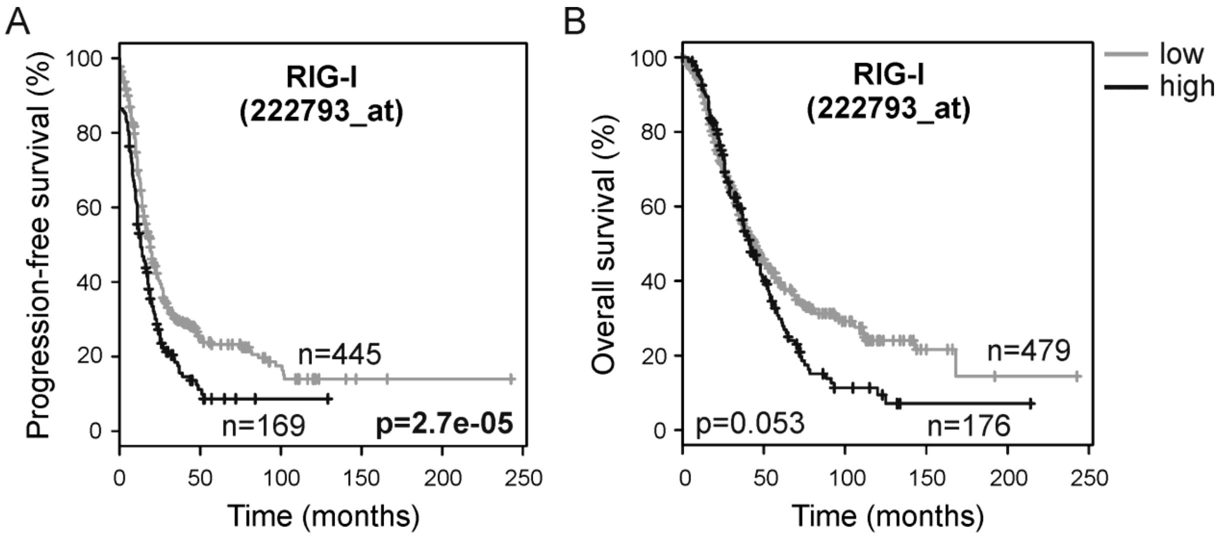

Supplement: Supplementary file 1 — Appendix S1: Supporting Information [file IJC-146-2007-s001.pdf]
